# Why do we still need participatory technology assessment?

**DOI:** 10.1007/s10202-012-0122-5

**Published:** 2012-11-13

**Authors:** Leonhard Hennen

**Affiliations:** Institute for Technology Assessment and Systems Analysis, Karlsruhe Institute of Technology, c/o Helmholtz-Gemeinschaft Ahrstr. 45, 53175 Bonn, Germany

## Abstract

The paper contributes to the current discussion on the role of participatory methods in the context of technology assessment (TA) and science and technology (S&T) governance. It is argued that TA has to be understood as a form of democratic policy consulting in the sense of the Habermasian model of a “pragmatist” relation of science and politics. This notion implies that public participation is an indispensable element of TA in the context of policy advice. Against this background, participatory TA (pTA) is defended against recent criticism of procedures of lay participation which states that pTA is lacking impact on S&T decision making, that pTA instead of opening S&T policies to new perspectives is used as a means to support mainstream S&T policy and that in pTA procedure the authentic lay perspective is systematically contorted by dominant expert knowledge.

## Abstract (de)

Der Aufsatz befasst sich mit der spezifischen Rolle partizipativer Verfahren im Kontext von Technikfolgenabschätzung (TA) und Technology Governance. Es wird argumentiert, dass Technikfolgenabschätzung als Form demokratischer Politikberatung im Sinne des von Jürgen Habermas als “pragmatistisch” bezeichneten Modells gemeinsamer Beratung von Wissenschaft und Politik verstanden werden muss. Diese Auffassung impliziert, dass die Beteiligung der Öffentlichkeit ein notwendiges und unverzichtbares Element politikberatender TA ist. Vor diesem Hintergrund verteidigt der Aufsatz partizipative TA gegen einige zentrale Motive der Kritik partizipativer Ansätze in der Technology Governance, die auf den mangelnden Einfluss solcher Verfahren auf technologiepolitische Entscheidungen, auf die Instrumentalisierung von Partizipation zur Unterstützung des wissenschafts- und technologiepolitischen Mainstreams sowie auf die angebliche Verfälschung der Laienperspektive durch die Dominanz von Expertenwissen abstellt.

## Résumé

L’article discute le rôle particulier des processus participatifs lors de l’évaluation des conséquences technologiques et la gouvernance technologique. L’argumentation est la suivante: l’évaluation des conséquences technologiques doit être comprise comme un mode de consultation politique démocratique au sens du modèle de consultation commune des cercles scienctifiques et politiques, caractérisée jadis de “pragmatistique” par Jürgen Habermas. Ainsi, la participation du public dans l’évaluation des conséquences technologiques est entendue comme élément indispensable aux délibérations politiques dans ce domaine. Dans ce contexte, le présent article défend l’évaluation des conséquences technologiques participatives contre certains motifs centraux de criticisme d’approches participatives en gouvernance technologique mettant en relief le manque d’influence de tels processus sur les décisions prises en politique technologique, l’instrumentalisation de la participation pour soutenir plutôt le courant dominant des politiques scientifique et technologique ainsi que la falsification prétendue de la perspective laïque par l’influence prédominante de l’expertise.

## Introduction

After more than 20 years of practice of and theoretical and methodological discussion on participatory methods in the context of science and technology (S&T) governance, we on the one hand still observe an increased interest in participatory events in S&T policy making. On the other hand, there is significant sobering as regards high flying expectations with respect to democratising S&T policy making via participatory technology assessment (pTA) and the function and role of participatory methods. Also, the “discovery” of participatory TA as an object of research for social studies of science and technology has led to a critical debate on participatory TA in the social sciences.

Starting from a reflection on the mission and role of TA as a model of policy consulting and advice that is led by the classical text of Jürgen Habermas on what he calls a “pragmatist model of the relation of science and politics,” I undertake to assess the findings and arguments of recent critical work on participatory TA procedures and discuss three motifs of criticism regarding: (a) the unclear contribution of participatory TA to the policy-making process, (b) its instrumentalisation as a means of raising public acceptance in mainstream S&T policies, and (c) its inherent tendency of subduing or blurring the authentic perspective of laypeople by expert rationality.

Notwithstanding the fact that the criticism duly highlights weaknesses of participatory TA and problems of its function in and relation to established modes of S&T policy making, the paper argues that the criticism insufficiently takes into account the context of participatory TA as an element of policy consulting. By this, it overstates the mission of pTA in terms of its political impact (3.1) and tends to inadequately conceive of pTA as a means of raising public support for mainstream S&T politics by symbolic public engagement (3.2). When taking into account the sociological concept of the layperson and the expert as being complementary social roles (3.3), it becomes clear that it is mainly a misconception of the relation of lay and expert knowledge that leads to wrong expectations with regard to the role of laypeople and lay knowledge in participatory TA.

## Technology assessment’s self-conception

Since its beginnings, technology assessment (TA) has been understood to be a procedure of scientific policy consultation that responds to the problems posed by two ideal type models of the relationship between scientific expertise and political decision making. Both of these ideal types are ultimately based on an (illusory) separation of facts and values in political decision-making processes. In his essay “The Scientization of Politics and Public Opinion” (1971; first published as “Verwissenschaftlichte Politik und öffentliche Meinung” 1963/1968), a classic text for TA (at least in Germany), Jürgen Habermas refers to these types as “decisionistic” and “technocratic.”

In the *decisionistic* model, policy makers in politics have scientists who inform them about the means made available by technology, yet ultimately it is on the basis of power and interests that the policy makers determine the goals (values) for which the means are employed. In this sense, science is, as it were, politically instrumentalized. In contrast to that model of political sovereignty, the *technocratic* model can be viewed as one that takes into account the growing societal significance of science and technology and the scientific rationalization of everyday life and politics. Here, all political issues are ultimately reduced to factual ones. They are not decisions about values. On the contrary, the assumption is that all decision making issues can in the final analysis be resolved on the basis of scientific–technological rationality. Political debates are ultimately replaced by expertise.

It is possible to view the entire development of the discussion in the social sciences and philosophy (starting from the 1970s) about the societal role of science and technology, the “seamless web” of science and technology (Hughes)—that is, in principle what we refer to as modern social scientific research on technology and as the philosophy of technology—as a refutation of the fundamental assumption underlying both models.

As early as 1963, Habermas pointed out that both models are based on untenable fundamental assumptions. They not only are an inadequate description of the actual practice of scientific policy consultation but also from a democratic perspective both inappropriate and impracticable. He proposed in contrast a “pragmatist” model that, in my opinion, describes the actual claim and self-conception of TA as policy consultation.

The outcome of this model is, in summary, that normative claims (values and needs) have to be examined with regard to their generalizability, feasibility, costs, and utility in the light of scientific and technological knowledge. Conversely, scientific and technological knowledge (of means) has to be assessed in the light of normative and evaluative standpoints. This pragmatic discourse between politics and science is always dependent on an informed public debate. The discourse about a rational choice of social goals and means can only be conducted *with* public participation—instead of it—if the decision-making procedure is not supposed to derail in a decisionistic or a technocratic manner with well-known “unpleasant” consequences for science and politics themselves, such as problems of acceptance and legitimacy.

Core elements of the pragmatist model are that scientific–technological knowledge is accessible and transparent to both the public and policy makers as well as—as one would say today—that politics and science are “responsive” to the demands formulated by an enlightened public debate. Technology assessment has always been located at precisely this position as a consequence of its assignment to inform policy makers in an independent manner about scientific and technological issues and their social implications as well as about the anxieties and expectations formulated in society. This can be traced back to the debates surrounding the establishment of the Office of Technology Assessment at the US congress in the 1960s (Hill 1997; Guston and Bimber 2000).^1^ Even though the significance and role of participatory procedures vary from institution to institution and according to political culture, the topic of public involvement—hence, participation in consultation and policy analysis (the expression “participator policy analysis” has been used in this regard)—is necessarily a general topic for TA. Without (some kind of) participation, there can be no TA. TA without participation is at least in danger of missing this goal of its public assignment and its functionality with regard to society’s capability of dealing with scientifically and technological-induced “uncertainties” and “ambiguities” (cf. Hennen 1999).

Participation is thus not simply some arbitrary method for TA but an essential part of its conception. The problem with participation is the fact that it—as an attempt to implement or step toward democratic governance of technology policy—first has to create the conditions for its own existence. Habermas noted long ago that essential “empirical conditions for the application of the pragmatist model are lacking,” including enlightened public debate. Experts and political decision makers are, finally, holding firmly onto a “bureaucratized exercise of power.” This, in Habermas’ words, “has its counterpart in a public realm confined to spectacles and acclamation. This takes care of the approval of a mediatized population (Habermas 1971, pp. 75f. Habermas 1968, p. 139). He, nonetheless, not only holds on to this—democratic, as it were—ideal of policy consultation, but also views this model “as a regulative idea,” still at work, although it has never become reality in a practical political sense. This leads to the problem with participatory TA: It will never be possible for it to become reality in accordance with its high aspirations in innovation and market societies (which Habermas referred to as “late capitalism”).

It is—according to Habermas at that time—decisive for a democratic rationalization of political decisions on issues of technology policy “whether a productive body of knowledge is merely transmitted to men engaged in technical manipulation for purposes of control or is simultaneously appropriated as the linguistic possession of communicating individuals” (Habermas 1971, p. 79/Habermas 1968, p. 144). In other words, it is decisive that the expert and the layperson (keyword: layperson participation) are in consultation with each other. I will return to this below when I confront several motifs of the criticism of participatory TA.

## Criticism of participatory TA procedures

For a long time, critical assessment of participatory TA was limited to evaluation of the process with regard to whether it met the procedural standards (fairness, transparency, etc.) that it itself had set and to whether the process enabled the participating laypeople to comprehend complex scientific–technological facts (expert knowledge) in a manner that enabled them to develop a position of their own on the related issues concerning the social–political consequences and regulation. A series of concomitant studies have shown that laypeople are in fact capable of dealing with expert knowledge in a rational manner and of reflecting on and grounding their own standpoint on ethical issues in the light of this knowledge (Mayer et al. 1995; Enderlin-Cavigelli and Schild 1998; Mørkrid 2001; Zimmer 2002). They also showed that the attitudes of laypeople can (but do not have to) change in the course of a participation process, that is, that a learning process is possible with regard to an individual’s own attitude toward ethical or risk issues. From a different perspective, participatory TA procedures have been examined with regard to the role that they play in different institutional, political, and other social contexts (Joss and Bellucci 2002). These approaches may truly be considered as self-reflection by the participating TA researchers (or of the organizers of participatory TA), so to speak as from an insider’s perspective. This perspective may be constrained by its own blind spots, but may also demonstrate some advantages versus the perspective of an outsider, such as with regard to the access to information or the use of realistic standards that are appropriate to one’s own activity.

For sometime now, participatory procedures concerning issues of science and technology policy have attracted the interest of the STS community. This produces an outsider’s view of participatory procedures, which on the one hand can result in insights that could not be reached from an insider’s perspective, but which also introduces demands that refer to democratic participation in science and technology policy in general without adequately taking into account the specific role that participation plays in the context of TA.

The critical appraisal that is presented in these studies is partly based on more general reasoning on the political role and function of participatory TA procedures (Levidow and Marris 2001; Abels 2002; Rayner 2003; Stirling 2007; Abels 2007; Wynne 2008). More recent criticism is based on case studies of single participatory processes with more or less explicit conclusions from the single case toward a general appraisal of participatory TA’s role (Bora and Hausendorf 2006; Felt and Fochler 2008; Bogner 2010; Felt and Fochler 2010; Degelsegger and Torgersen 2011). It is impossible here to follow the single lines of argumentation based in diverse theoretical backgrounds laid out in these papers. In the following, I want to put forward a few arguments against the guiding lines of criticism that can be found in this literature. There are primarily three motifs that are made apparent by summarizing the objections and criticism raised in various general reflections on participatory TA or in case studies on individual participatory TA proceedings:

- Participatory TA does not produce anything: the political role of such procedures in unclear, and their effect on political decision making is marginal or even completely indiscernible.
- Participatory TA is being misused: The increasing use of participatory procedures in science and technology policy is not an indicator of a new understanding of “technological citizenship,” an extension of citizens’ rights in science and technology policy making (Frankenfeld 1992). On the contrary, participation is instrumentalized to push through specific goals in innovation policy.
- Participatory TA contradicts the actual goals of participation: A layperson’s rationality does not have a chance against expert knowledge and the formalized decision-making procedures aligned on factual reasoning. In other words, the authentic perspective of a layperson is systematically distorted in such procedures.

### Lack of impact

The direct and visible influence of participatory procedures on political decision making is usually minimal and hardly apparent. Yet participatory TA procedures share this fate with forms of independent scientific consultation, as we know from research on the use of scientific knowledge in consultations (Albaek 1995; Hoppe 2005).

The individual processes of scientific consultation are part of a broad range of public debates, informal consultations in political parties and organizations and formal consultations of the actors involved by scientific experts. In this landscape, it is often impossible to follow the trail of individual consultation procedures and their outcomes. It is, moreover, the painful experience of everyone who practices TA as policy consultation (e.g., in parliamentary TA) that, as a rule, it is the exception for TA to produce a visible manifestation such as in parliamentary consultations, not to mention for the results of TA to be transferred into political decisions. Most results of scientific work produce, at the most, helpful support in political decision-making processes that takes the form of background knowledge of individual representatives. There are numerous reasons for this in individual cases. One systematic reason is, for example, that *independent consultation*, such as is provided by TA, is—in contrast to *mandated science*—not in a position to directly serve the immediate interests of specific actors. From the perspective of decision makers, the knowledge made available by TA raises the complexity of a decision-making situation rather than reducing it.

Thus, we should not set the bar any higher for participatory TA than for independent scientific consultation in general. My recommendation is that we—when looking at the impact—should focus less on the direct influence on decisions than—depending on the complexity and numerous facets of political decision making—on the resonance or visibility of TA or participatory TA in political debates.

The comparison of 16 participatory TA procedures that was conducted in the context of the EUROPTA study showed that in particular two factors are decisive for the visibility and resonance of participatory TA (Hennen 2002): *the character and status of the public debate* and *the institutional and political setting of the procedure* (especially the nature of the assignment and the standing of the institutions carrying out the TA and the relationship of the procedure to the formal structures of political decision making).

The chances for participatory TA to find resonance in the debates, that is, for individual actors to pick up on certain results, are rather small if a debate over technology is well advanced, if individual interest groups have already taken firm positions, and if political decisions are pending short term. The chances were relatively good, however, for topics on which different actors had not yet taken a position, which concerned conceptional issues of formulating policy, and for which there had been a relatively open search for ideas and descriptions of the problem. In that period, that is, at the end of the 1990s, this was the case for, above all, the topic of sustainable development.

The second factor concerns the procedure’s standing or the established political decision-making structures facing the institution conducting the procedure. A fundamental finding was that procedures that did not have an institutional connection to the decisions—such as because they were conducted by academic institutions at their own initiative—produced correspondingly little resonance. Resonance could be achieved where the institution responsible played a defined role as a consulting institute (such as of parliament) and where furthermore public involvement belonged to its political culture (which implies in particular a certain amount of commitment of the political institutions to the procedures). This was true, for example, for the procedures conducted by the Rathenau Institute (The Netherlands), but in particular for those conducted by the Danish Board of Technology (DBT).

With regard to several of the recently published critical case studies on participatory TA procedures, I would like to point out that the procedures studied there are often precisely those—to consider their institutional setting—that were not situated in the context of policy consultation conducted by an institution working with a public mandate. The procedures studied were, on the contrary, rather free floating, conducted on unclear assignments, and performed by “participation entrepreneurs” (Bogner 2010).

Yet it cannot be denied that (also for TA carried out in a context of policy consulting) the role of participatory procedures is often unclear toward the established decision-making structures, also that such procedures are not anchored in the political culture and that a corresponding commitment by political institutions is lacking. Thus, essential boundary conditions in this case are quite bad, which explains the difficulties of participatory TA to produce resonance. Participation in the context of TA is in essence a method for identifying the perspectives and aspirations of social groups, but can by itself not produce any public or political resonance. The proximity to structures of political decision making can, however, lead to keeping participatory TA from coming into play or from having it perceived as an irritation or a danger.^2^

In view of these problems, it is, however, wise not to throw out the baby with the water. Participation still has a role to play in the context of a TA study even if, for example, there is no immediate resonance in the media. Modifications in the conditions of a context can be accompanied by displacements in the political structure and culture and open opportunities for resonance in individual cases. It is after all a part of the activity of policy-consulting TA for it to create attention or resonance for the results of its own work, something that happens on its own in the rarest of cases.

### Instrumentalisation

The in many cases, unclear institutional role and the unclear political assignment of participatory TA raise suspicions that it serves solely as an instrument of symbolic politics and as a means to force through goals and strategies for innovation and technology policy. It then does not appear to represent a serious attempt to introduce an element of public participation or technological citizenship into established, expert-centered decision-making structures. A risk associated with the latter would be an unpredictable outcome that might appear unpleasant to the decision maker.

This criticism has been directed strongly, for example, at the strategy variously propagated by the EU since the White Paper on Governance (European Commission 2001a, b) of moving toward new forms of governance (including in the area of technology policy) with an emphasis on involving European citizens (Levidow and Marris 2001; Abels 2002, 2007). According to this criticism, the new forms of governance propagated since the Science and Society Action Plan (European Commission 2001c)—for which the EC has employed expressions such as “socially robust knowledge,” “democratizing expertise,” “informed participation by society in policy making,” and “particularly in defining the priorities of publicly funded research”—are nothing more than rhetoric (“rhetoric of openness,” Levidow and Marris 2001). For these critics, participatory procedures are not meant to establish new contact between science and society that opens technology policy for new ideas and influence but solely to reestablish confidence in the existing institutions in order to continue pursuing the old goals of innovation policy. I do not want in any manner to deny that the critics correctly contrast programmatic announcements and the modest practice of reality. Yet something here appears to me to be symptomatic for many critical comments about participatory TA. This is the overloading of participatory TA with expectations of a so to speak, completely direct democratic transformation of the institutions and decision-making processes in technology policy. It is obvious, after all, that new participatory elements of governance will not throw the classical goals of technology policy overboard.

The goals that continue to determine the strategy to create a European research area, in the context of which new forms of governance are also called for, are the strengthening of the EU in international technological competition and the increased utilization of innovations to achieve economic growth in Europe. In practice, participation as a form of governance is naturally conceptualized as invited participation, not as mobilization of protest. Citizen involvement will continue to be subordinate to the goals of innovation and economic policy. Yet just as for example, the precautionary principle in the practice of the European Commission by all means implies criteria of good governance and these criteria are at odds with the innovation policy agenda to strengthen the EU in global competition, so too does the voluntary obligation to support “participatory policy making” produce some democratic friction. It is not the case that the rhetoric has been entirely without consequences. Let me simply refer to the support for “science in society” and for research on ethical, legal, and social aspects in the framework program and above all to the practice of resorting to public consultation in the commission’s formulation of policy and its regulatory practice.

It cannot be denied that participatory procedures are in danger of being instrumentalized and manipulated. Andrew Stirling (2007) has pointed out that every type of appraisal of technologies made in the context of policy consultation (whether by experts or through participation) is in danger of being instrumentalized by power and justification strategies. In other words, TA can be framed, conceived, and even controlled from the outset so that it does not pursue the purpose of consultation (opening the decision-making situation for new facts, interests, and values, for example) but is laid out to close debates over technology and to use TA to legitimate certain preferred strategies or options for action. Scientific or expert appraisal (expert bodies, advisory councils, and commissions) can be controlled, for example, by means of assigning priority to certain research issues, of the accreditation of experts, and of selecting the members of councils. Similarly, there are multiple opportunities to steer participatory procedures to the desired results by means of the procedure’s design, the selection of the participants and the topics, the structuring of the issues, and the moderation of the discussion and the consultations. Stirling (2007, p. 277) comes to the conclusion, “It seems clear that the apparent normative democratic credentials of participatory appraisal do not themselves confer immunity to instrumental pressures for the justification of powerful interest.”

I would assume that every organizer of a participatory procedure would emphasize this judgment. Yet it should be added that there are rules of good project management and design for preventing precisely this from happening. Everybody knows about scientific fraud and cases of lip service of experts, but nevertheless only few would assert that therefore the rules of scientific activity such as peer review or the Mertonian norms of science (communalism, universalism, disinterestedness, originality, skepticism) are ineffectual and that science is therefore an obscure undertaking. Yet just as little should the fact that manipulation of participatory procedures does occur lead anyone to draw the conclusion that the rules of good participatory practice (which ultimately are based on the principles of discourse ethics) are senseless and ineffectual. It is precisely this problem which shows how important the institutional setting of the responsible organization is. It makes a difference whether a ministry commissions a participation contractor or brings an institution into play that is committed to independence as shown by its mission statement. I would therefore like to recommend that studies on participatory TA distinguish between the shortcomings of project management and the structural limits or deficits of the participatory procedure itself.

As a final comment on the topic of instrumentalization, it is worth noting that the literature on participatory TA contains examples for the fact that layperson consultations produce results that are opposed to the expectations or wishes of scientific and political elites (examples in Joss and Bellucci 2002; Schicktanz and Naumann 2003). I refer here for instance to the results published by (Dryzek et al. 2009) of a study of seven citizen juries and conferences on the topic of genetically modified food. The study showed that the comments of laypeople diverged in every case from the expectations of the interested parties. Proactive risk management and precaution were the central topics at each of these consultations and shaped their demand for restrictive regulation that was at odds with views dominated by opening up innovative pathways held by representatives of science and industry.

### The irreconcilability of layperson and scientific reasoning

By asking the question, “What happens with the layperson’s perspective in participatory procedures?,” I come to a point that is specific to the criticism of procedures that attempt to involve citizens as representatives of the public in general. The criticism that the rationality or perspective of laypeople in procedures based on deliberation is systematically subject to force is grounded in different ways. I think that a problem of this criticism is that it is not clear what this specifically layperson’s perspective actually consists in. It appears to be subliminally linked with a romantic idea of the authenticity of the “lay perspective”.

The argument is still quite clear and plausible if a layperson’s perspective interested in issues of justice and in values of a good life confronts the rationality of administrative, formalized procedures for evaluating risk. In a study of authorization procedures with citizen involvement that concerned the release of genetically modified organisms, Bora and Hausendorf have shown how these formalized legal procedures systematically exclude core concerns of laypeople. The value-oriented arguments of citizens do not fit into the procedure’s scheme designed to process factual issues of scientific risk evaluation. The narrow definition of “technological citizenship”—as something whose content is related to scientific issues of truth—that the procedure prescribes is forced through, systematically disappointing the understanding of technological citizenship held by the participating citizens as extended civil rights in technology policy.

This might in fact be true in the case of a formal administrative authorization procedure that has to conform to certain legal constraints. Another definition of citizenship should, however, be possible in the context of technology assessment as policy consultation. Technology assessment is not subject to the pressure of utilizing a legally watertight procedure to reach incontestable decisions, but aims at preparing a policy analysis that is as comprehensive as possible prior to decision making. In this case, the rationality of political decision making should be more comprehensive than scientific risk assessment.

Yet there is another form of the criticism that an authentic layperson’s perspective would systematically fall victim to the expert’s perspective in the case of participatory procedures on scientific–technological issues. I do not want to dispute that the narrow phrasing of an issue can lead to certain arguments being excluded. It also may happen that, as mentioned in one case study (Felt and Fochler 2010), the pressure to have to (or want to) come up with a presentable (sensible) product in the form of a citizens’ vote at the end of a procedure can seduce the organizers and moderator to streamline the group’s discussion. What I said about instrumentalization above applies to these cases as well. The critical analysis wants to say more than this, however, namely that the layperson’s perspective does not get a chance in the communication between experts and laypeople or is twisted (Felt and Fochler 2008, 2010). This is supposed to prevent precisely what participatory TA wants, that is, giving the layperson’s perspective a chance.

To engage this criticism, it is helpful to recall a few insights into the concepts “expert” and “layperson” (Sprondel 1979; Schütz 1946, cf. also Hennen 1992, p. 190ff.). Layperson and expert are, as is well known, complementary roles: without experts, no laypeople, and vice versa. They are formed in the course of the processes of scientization and technicalization, without which there would neither be experts nor laypersons. In the course of this technicalization and scientization, people become dependent on experts in more and more areas of life. This means, on the one hand, the removal of an enormous burden from laypeople and an extension of their options to act (generally also known as the blessing of technological progress). The flipside of this is a systematic “minimization of know-how” (Linde 1982; Hennen 1992, p. 175ff.) or a loss of control over action on the side of the layperson. A large portion of the control and responsibility for the functioning of daily life has, so to speak, been delegated to experts. This delegation comprises the entire scope of daily live from the guarantee that technological equipment and the infrastructure are in working order to—for instance—family planning, as shown by the visits to a human geneticist or specialist in reproductive medicine.

If we take the complementarity of experts and laypeople seriously, then we must reach the conclusion that the layperson’s perspective can only be formulated in relation to expert knowledge. This—poignantly phrased—would imply that the most “authentic” layperson’s perspective—as often demanded in the discourse on participatory TA—would be a perspective of not having a clue (which would imply complete dependence on experts).

Always at issue in all the debates about the governance of science and technology and about science in society, as well as in all conflicts over technology are ultimately problems that result from a specialized system of experts continuously generating innovations. These innovations want to be implemented socially and in the process encounter laypeople who—allegedly because of a lack of knowledge and/or the retention of certain values—are somehow unwilling to warm to the offers being made. They know on the basis of past experience, furthermore, that you cannot have innovation without risk and without destroying existing structures (i.e., it always damages certain values and interests). And it is also scientifically impossible to predict with certainty which effects innovations will have in the long run. All of this leads to well-known phenomena such as, for example, ethical debates and problems of acceptance.

The aim of participatory procedures for new technologies can, in my opinion, only be to put laypeople in a position to make an informed opinion about each new achievement of expert knowledge. This is necessarily associated with an element of learning or empowerment. This is an advantage of participatory procedures compared with standard instruments of surveying public opinion. Opinion polls on new technologies are usually conducted with participants in a state of ignorance. The distribution of answers can then be considered statistically representative, yet these are the answers of people who possibly had previously never given any consideration to the topic in question. Participatory TA cannot provide representativeness of that sort. Its goal is to obtain a layperson’s judgment based on knowledge, simulating informed public opinion. In a certain sense, this is what is demanded in the pragmatist model of policy consultation as the sine qua non of rational democratic decision making, but that—even in Habermas’ view—is at most present in a rudimentary form in the reality of mass and media democracies.

This means that an authentic layperson’s perspective—in the sense of an informed citizen—can only be formed in the course of confronting expert knowledge. Evans and Plows (2007) attempt to determine the specific knowledge that laypeople can contribute to technology appraisal is much in line with Alfred Schütz’ (1946) concept of the “well informed citizen” as holding a non-specific and therefore ubiquitous perspective on problems of everyday life, that discerns the informed citizen from the specialized but restricted expert’s perspective. For Evans and Plows, a true layperson is characterized by not disposing of specific knowledge but only of “ubiquitous” or “popular” knowledge. Their disinterestedness or impartiality (their “being not involved”) is regarded by Evans and Plows as the central feature that enables laypeople to develop an “external meta perspective” when dealing with contested issues of science and technology. When properly informed, they are able to appraise expert knowledge with regard to its practical (everyday life) implications. The appraisal is based either on general social beliefs (“ubiquitous discrimination”) or on particular knowledge and beliefs that they hold as a member of a particular community (“local discrimination”) (Evans and Plows, 831). The lack of a specific commitment enables laypeople to act as an independent jury, hearing evidence from different experts, and knowledge cultures. “Only those who are situated outside the committed knowledge cultures of both the scientific and activist communities can bring in a genuine civic epistemology” (Evans and Plows 2007, 843). I admit that more research is needed in order to understand the genuine perspective of laypeople, but it is evident that this perspective can necessarily only evolve in an (as much as possible) independent appraisal of expert and stakeholder knowledge.^3^

Summing up on the argument of the endangered state of the authentic layperson’s perspective in pTA I would conclude: If prejudices are given up in the course of a participatory process or if certain views and expectations do not survive or are discarded in the course of the consultation, this does not necessarily indicate an undue deformation of the “authentic” lay perspective. Yet the discursive elimination of perspectives that are naïve, inadequately grounded, or implausible is a fundamental feature of the procedure. And even if—as can be read in a case study from Austria (Felt and Fochler 2008)—a panel of laypeople reflects on its own role in the course of a procedure and reaches the conclusion that it could not really speak in the name of the general public because the panel’s members have a significant advantage over the public in terms of knowledge, this is not a deficit of the procedure (because it fails to represent the opinion of the general public). It is, on the contrary, proved that the participating citizens have understood their role quite well.

## Conclusions

Participatory TA should fundamentally be considered an element of what Sheila Jasanoff (2005) has termed “civic epistemology.” The emphasis should be on “an element,” namely as an element of the manner in which societies adapt and value scientific knowledge. In this regard, it is one element among several that are decisive, some of the others being the forms of political representation, the role of experts in the political system, the culture of public deliberation, and the degree of transparency in public institutions. It is a deliberative element of the organization of the way in which society treats knowledge, not an Aristotelian point to turn the system upside down.

Participatory TA should not be overloaded with unrealistic expectations. Participation plays a role in the context of TA performed as policy consultation and is thus not political participation in decision making but participation in ascertaining the available knowledge and evaluating it in the light of social values and interests. Certain freedoms are linked with this which are missing in civic participation in formal decision-making processes. The price of this freedom is the relatively non-binding nature of participation. The purely informal role of citizens makes TA open for a broad spectrum of opinions and demands. Yet this is also tied to the problem that participatory procedures inevitably generate expectations regarding a resonance in representative political decision-making systems. An obvious reaction to the criticism that there is insufficient resonance would be to limit the role of participation in the context of TA a priori to that of an instrument from the social sciences for gathering knowledge. Taking into account the claims that result for the public from the pragmatist model of policy consulting, I do not hold this reaction to be desirable. The context of policy consultation—which is the consultation related to decision making that is open to public view, with the claim to transparency and normative inclusiveness—necessarily implies three types of expectations:

1. Participatory TA is a qualitative (scientific) method for determining the attitudes, interests, and patterns of argumentation used by laypersons with regard to complex issues of science and technology policy. Participatory technology assessment in this regard is supposed to improve the knowledge basis of policy decisions.
2. As mini-publics committed to deliberation, TA procedures have to position themselves as an element of the general public sphere, to be accessible to public observation, and to stimulate and inform public debates about technology. In other words, it contributes to an informed public discourse.
3. Participatory TA will thus always see itself exposed to the expectation of improving the responsiveness of the political system and of giving a voice to perspectives that are not or only poorly represented in political debates and decision-making processes.

What is applicable here, just as it is for the pragmatist model of policy consultation in general, is that TA as an element of democratic “civic epistemologies” has to hold its ground against other interests, structures, and strategies. Thus, TA must sort of “co-produce” the conditions of its contribution to the societal process of dealing with uncertainty. It can only approach its goals gradually, disturbed by manifold types of interference with social reality.

## Footnotes

1 Other than is often purported in discussions about the origins of the OTA the foundation of the office by no means was alone due to an expressed need of arming the US congress with expert knowledge in view of the increasing power of the science and technology department of the executive branch. Guston and Bimber show that concomitant with a discourse on the need for unbiased analysis mainly maintained by scientists and parliamentarians there was a discourse on the integration of citizens’ perspectives in S&T policy making that was behind initiatives to establish the OTA. Referring to this participatory discourse, Guston and Bimber (2000), p. 5f.) even speak of a “social movement akin to environmentalism and consumerism.”

2 The author can provide anecdotic evidence on this from two unsuccessful attempts to conduct participatory TA at the Office of Technology Assessment at the German parliament.

3 Evans and Plow’s plea for fostering the role of the citizen indicates at least the direction that the further exploration of the contents and structures of civic epistemologies and laypersons contribution to it should take. A layperson’s “knowledge system,” as any, comprises not only manifest inventories of knowledge but also methods and beliefs for selecting the relevant from irrelevant, the true from the false, etc. For a proper analysis, thus, reference to concepts of everyday knowledge and orientation will be needed (making use of theories of everyday life as provided by, for example, Alfred Schütz or ethnomethodology) as well as a differentiated analysis of lay knowledge in relation to different types of problems and issues that are dealt with in pTA.
